# Rapid Generation of Leukemogenic Chromosomal Translocations in Vivo Using CRISPR/Cas9

**DOI:** 10.1097/HS9.0000000000000456

**Published:** 2020-09-30

**Authors:** Yun Huang, Blerim Marovca, Susanne Dettwiler, Peter Karl Bode, Beat Bornhauser, Jean-Pierre Bourquin

**Affiliations:** 1 Pediatric Oncology, Children's Research Centre, University Children's Hospital Zurich, Switzerland; 2 Institute of Pathology and Molecular Pathology, University Hospital Zurich, Switzerland.

A wide range of chromosomal rearrangements have been identified in human cancers especially in leukemia.^1,2^ Murine models that recapitulate chromosomal translocations are essential for understanding the determinants of malignancies and as a basis for preclinical development of rational therapeutic methods. Genetically engineered murine models involving chromosomal translocation genes have been generated using transgenic approaches or gene knockin.^3,4^ However, the closest model is the creation of bona fide chromosomal translocations, which recapitulate the physiologic levels of the fusion genes, the reduced dosage of the wild-type alleles and the contribution of the reciprocal products of the translocations. This has been achieved in vivo using the Cre-loxP system in embryonal stem cells,^5^ which is labor intensive and time consuming. The CRISPR/Cas9 system is a versatile tool for genomic engineering. Transduction of target cells with vectors encoding Cas9 and sgRNAs targeting two genomic loci causes two DNA double strand breaks (DSBs), which could induce chromosomal rearrangements as a result from non-homologous end joining-based DNA repair.^6^ This strategy has been used to model oncogenic chromosomal rearrangements in murine^7–10^ or human^11^ cells in vivo. However, given the challenges of delivering CRISPR/Cas9 vectors into hematopoietic cells, the penetrance of leukemogenesis has been shown with low frequency in vivo.^11^ Here we establish an efficient methodology to generate chromosomal translocations in mice hematopoietic cells based on a vector design enabling convenient lentiviral delivery of multiple sgRNAs to hematopoietic cells from a Cas9 expressing mouse strain.

The *KMT2A (MLL)* gene constitutes a hotspot for chromosomal translocations in human leukemia, resulting in over 135 different leukemia-associated *KMT2A* gene fusions.^2^*KMT2A-MLLT1* defines a leukemia subtype with poor outcome. This translocation frequently constitutes the sole genomic lesion to drive the leukemia,^12^ providing a good model to establish this approach. We designed a strategy to induce *Kmt2a-Mllt1* translocations in murine hematopoietic cells using the CRISPR/Cas9 system followed by transplantation into lethally irradiated recipient mice (Fig. 1A-B). Given the large size of Cas9, it is challenging to transduce murine hematopoietic cells with lentiviral vectors coding Cas9 together with sgRNAs. We therefore took advantage of the Cas9 knockin mouse,^13^ in which the Cas9 and a GFP reporter are expressed constitutively in the whole body. Using a single lentiviral vector to deliver two U6 promoter-sgRNA cassettes is an efficient way to induce two DSBs simultaneously.^14^ We constructed a lentiviral vector coding sgRNA and a fluorescent marker (sg_shuttle_RFP657, Supplementary Fig. 1A) with special designs: (1) The small size of the backbone with minimum interval sequences between each modules improves the virus titer; (2) The isocaudomer sites flanking the sgRNA cassettes facilitates the combination of multiple single sgRNAs from individual vector into one vector (Supplementary Fig. 1B). We firstly cloned several individual sgRNAs targeting the equivalent introns of *Kmt2a* and *Mllt1* that are orthologue regions in the human leukemia translocation (Fig. 1A-B). The editing efficiency of each sgRNA was firstly validated in NIH 3T3 cells stably expressing Cas9 (Supplementary Fig. 1C), then the most efficient sgRNAs targeting *Kmt2a* and *Mllt1* loci were combined into one vector (sg_ K-M). Transduction of this vector coding two sgRNAs in NIH 3T3 cells generated the *Kmt2a-Mllt1* translocation efficiently (Supplementary Fig. 1D).

**Figure 1:**
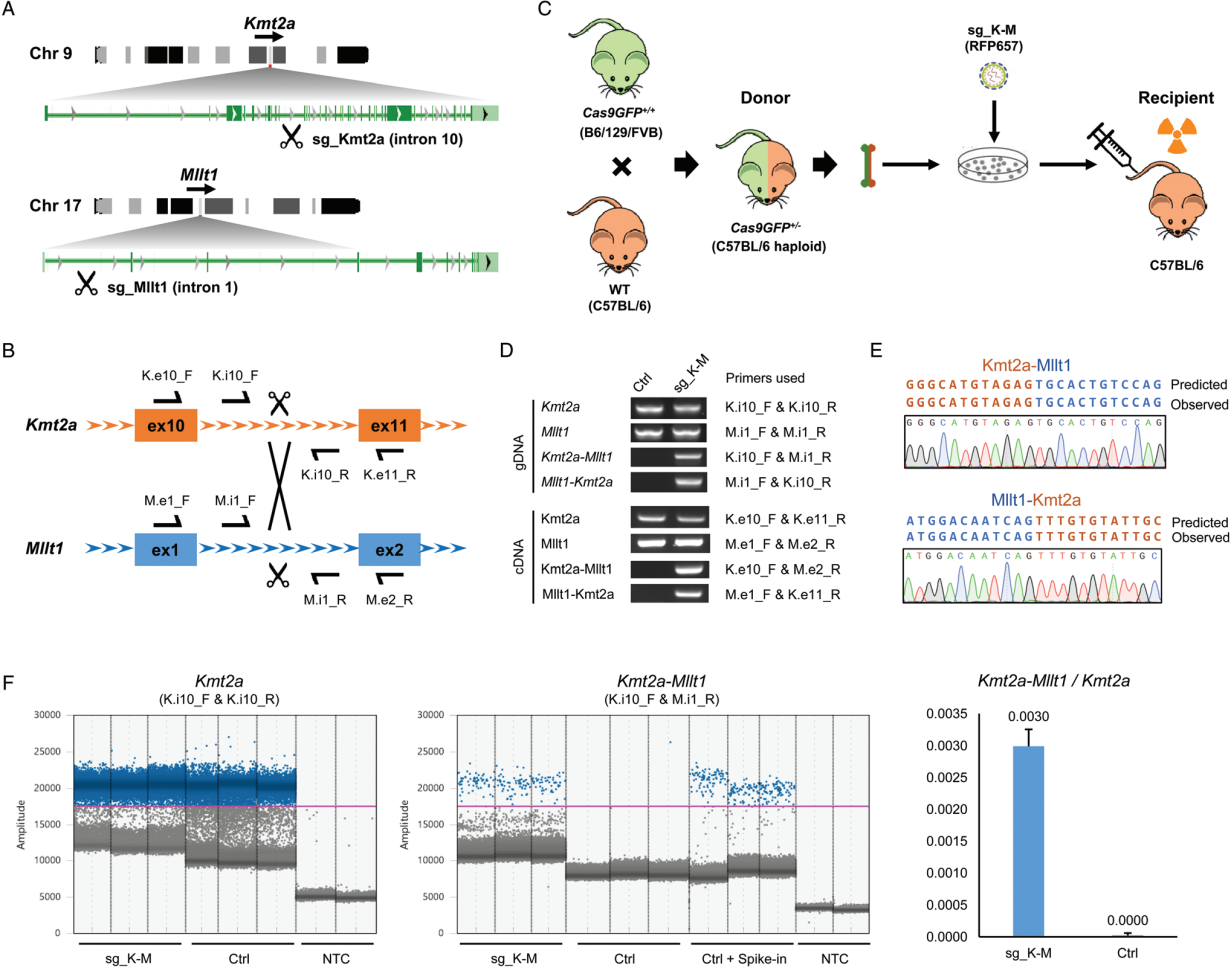
**Induction of the *Kmt2a-Mllt1* rearrangement in murine hematopoietic cells using the CRISPR/Cas9 system.** (A) Schematic of the t(9;17) involving the *Kmt2a* and *Mllt1* loci. Introns targeted by sgRNAs are indicated. (B) Schematic of the *Kmt2a* and *Mllt1* loci with the locations of the primers used for genotyping. (C) Strategy for the generation of the *Kmt2a-Mllt1* rearrangement in murine bone marrow cells and transplantation experiments. (D) PCR genotyping of cultured bone marrow cells from *Cas9GFP*^*+/−*^ mice transduced with or without sgRNA vectors (sg_K-M). PCRs were performed on genomic DNA (gDNA) or cDNA from bone marrow cells *ex vivo* cultured for 4 days after transduction. (E) Sequence of the PCR products from cDNA showing the Kmt2a-Mllt1 and Mllt1-Kmt2a junctions. (F) ddPCR quantification of *Kmt2a* and *Kmt2a-Mllt1* frequencies in gDNA from bone marrow cells ex vivo cultured for 4 days after transduction of sgRNA vectors. Signal amplitudes of amplicon-positive (blue) and -negative (gray) droplets for the detection of *Kmt2a* and *Kmt2a-Mllt1* (left and middle) and the copy number ratio of *Kmt2a-Mllt1* with respect to *Kmt2a* in Ctrl and sgRNA transduced groups (right, data are presented as mean with SD). Primer pairs for detections are indicated in the parenthesis; NTC, no template control; Ctrl+Spike-in, control gDNA spike-in with synthetic artificial DNA templates of *Kmt2a-Mllt1*.

We then applied this approach to target primary murine hematopoietic cells (Fig. 1C). Since the original Cas9 knockin mouse has a mixed background of B6/129/FVB,^13^ we crossbred the Cas9 mice with the inbred C57BL/6 mice and used the F1 progenies (*Cas9GFP*^*+/−*^) as the bone marrow donors. Given the C57BL/6 haploidentical setting, these cells can reconstitute the hematopoietic system in lethally irradiated C57BL/6 recipient mice without inducing graft-versus-host disease. Transduction of these bone marrow cells with the sgRNA vector (sg_ K-M) lead to a detectable genomic rearrangement between the *Kmt2a* and *Mllt1* locus at 4 days after transduction in ex vivo cultures (Fig. 1D). The expression of both Kmt2a-Mllt1 and the reciprocal Mllt1-Kmt2a fusions were detected by RT-PCR and verified by sequencing (Fig. 1D-E). Droplet Digital PCR (ddPCR) performed on genomic DNA from unsorted cells detected a copy number ratio of 0.003 of the *Kmt2a-Mllt1* with respect to the wild type *Kmt2a*, implicating that ∼0.6% of the cells harbor the translocations (Fig. 1F). Thus this strategy is fast and practical to generate de novo translocations in primary murine hematopoietic cells.

Transplantation of the engineered cells into lethally irradiated C57BL/6 mice lead to engraftment of a GFP^+^ population, which can be detected in peripheral blood at 4 weeks after transplantation. Three out of nine mice displayed a marked increasing of the RFP657^+^ populations in the myeloid population over time, which suggested the sgRNA-transduced population to become dominant in vivo (Fig. 2A). By 120 days after transplantation, 100% of the *Kmt2a-Mllt1* mice presented signs of sickness and were sacrificed (Fig. 2B). Post-mortem examination of *Kmt2a-Mllt1* mice confirmed the development of leukemia with predominant myeloblasts in the peripheral blood, splenomegaly and extensive infiltration of leukocytes into peripheral organs such as liver and kidneys (Fig. 2C and Supplementary Fig. 2). The immunophenotype of the bone marrow cells was consistent for a myeloid leukemia that was positive for myeloid markers (Mac-1^hi^, Gr-1^int^) but not for lymphoid markers (CD3, B220) or progenitor markers (Sca-1, c-Kit) (Fig. 2D).

**Figure 2:**
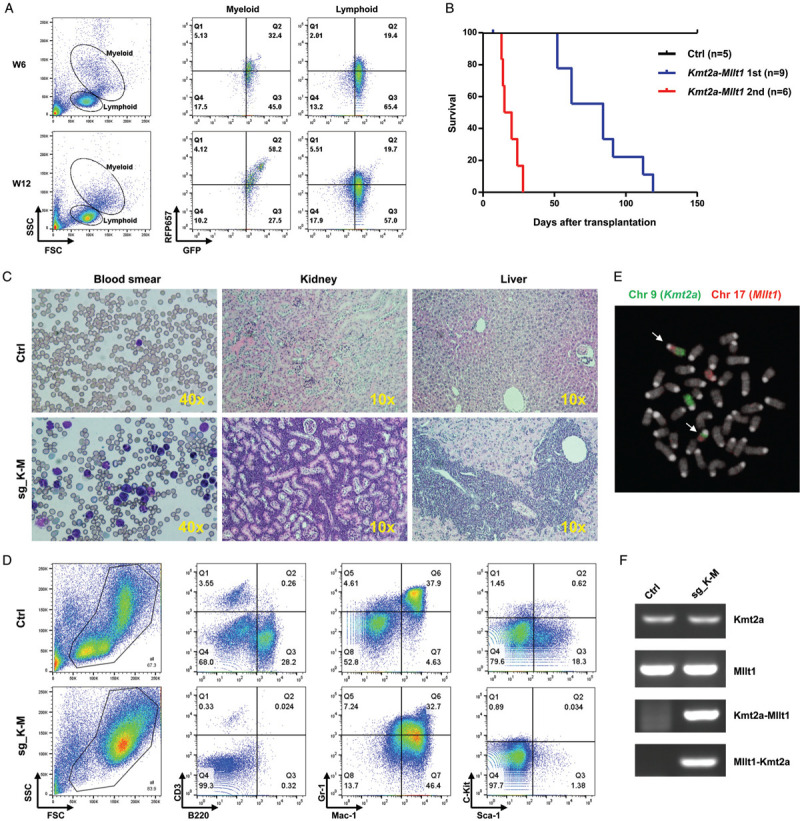
**The *Kmt2a-Mllt1* fusion induces acute myeloid leukemia.** (A) Flow cytometry analysis of peripheral blood cells from a recipient mouse transplanted with *Cas9GFP*^*+/−*^ bone marrow cells transduced with sg_K-M vectors at 6 weeks (W6) and 12 weeks (W12) after transplantation. A RFP657^+^ population with myeloid features is emerging over time. (B) Kaplan-Meier survival curves of mice transplanted with *Cas9GFP*^*+/−*^ bone marrow cells transduced with sg_K-M vectors developing primary (1st) and secondary (2nd) *Kmt2a-Mllt1* leukemia. (C) May-Grünwald Giemsa staining of blood smears and Hematoxylin-Eosin staining of tissue sections of kidneys and livers from control and *Kmt2a-Mllt1* leukemia mice. (D) Fluorescence in situ hybridization (FISH) on metaphases of splenocytes from *Kmt2a-Mllt1* leukemic mice using paints for chromosome 9 (FITC) or chromosome 17 (Cy3). White arrows indicate the translocated chromosomes. (E) Flow cytometry analysis of bone marrow cells stained with fluorescent antibodies in control and *Kmt2a-Mllt1* leukemic mice. (F) RT-PCR detection of the expression of the non-translocated alleles of *Kmt2a* and *Mllt1*, and the fusions of *Kmt2a-Mllt1* and *Mllt1-Kmt2a* in a control and a *Kmt2a-Mllt1* leukemic mouse.

The presence of the reciprocal translocations t(9;17) in leukemia cells was confirmed by FISH using pan-chromosome probes (Fig. 2E). The *Kmt2a-Mllt1* gene fusion and its product were verified by PCR and sequencing. Notably, both the non-rearranged allele of *Kmt2a* and *Mllt1* as well as the reciprocal product *Mllt1-Kmt2a* fusion were also expressed in the leukemia cells (Fig. 2F). Transplantation of the bone marrow cells from the primary tumor mice into lethally irradiated second recipients manifested diseases in a shorter latency of 2–4 weeks, confirms the self-renewal capacity and the aggressiveness of this leukemia (Fig. 2B).

We next attempted to model the *TCF3-HLF* translocation defining a highly aggressive leukemia subtype for which so far no representative mouse model could be engineered.^15^ Given the frequent *PAX5* heterozygous deletions and reduced gene expression identified in this leukemia,^16^ we tested the hypothesis if the *Tcf3-Hlf* translocation could induce a mouse leukemia when established in a Pax5 haploinsufficient context (Supplementary Fig. 3A–C). We crossbred the Cas9 mice with the *Pax5* heterozygous mice (*Pax5*^*+/−*^, C57BL/6 inbred)^17^ and used the F1 progenies (*Cas9GFP*^*+/−*^* Pax5*^*+/+*^ or *Cas9GFP*^*+/−*^* Pax5*^*+/−*^) as the bone marrow donors. Transduction of the bone marrow cells with validated sgRNAs targeting the equivalent introns of mice *Tcf3* and *Hlf* loci (sg_T-H) lead to detectable *Tcf3-Hlf* translocation and the expression of the fusion product (Supplementary Fig. 3D-E). The generation of the *Tcf3-Hlf* translocation was less effective than the *Kmt2a-Mllt1* translocation, with ∼0.16% of the cells detected by ddPCR to harbor the translocation (Supplementary Fig. 3F).

After transplantation of the engineered cells into lethally irradiated C57BL/6 recipients, the *Tcf3-Hlf* translocation was detected in genomic DNA from peripheral blood and bone marrow at 4 weeks after transplantation (Supplementary Fig. 3G-H). However, no progression to leukemia could be detected even after a follow-up for up to 11 months. Post-mortem examination did not reveal any abnormality of spleen, kidney, liver and bone marrow cells and the *Tcf3-Hlf* translocation signal was undetectable in bone marrow cells (Supplementary Fig. 3H). This suggests a selective disadvantage of cells carrying *Tcf3-Hlf* in vivo, confirming similar observations made with a knockin model of *Tcf3-Hlf*.^18^ Thus, despite the frequency of *PAX5* haploinsufficiency in *TCF3-HLF* positive patients, deletion of *Pax5* does not counteract the negative selection pressure by *Tcf3-Hlf*. Notably, the *Kmt2a-Mllt1* myeloid leukemia can be established in the *Pax5 *^*+/−*^ background with similar dynamics as in the *Pax5* ^+/+^ background confirming that a reduced *Pax5* gene dosage does not influence the leukemogenic activity of *Kmt2a-Mllt1* (Supplementary Fig. 4).

Activation of oncogenes by fusion or juxtaposition to ectopic regulatory elements constitutes important cancer initiating events. Here, we established an efficient strategy using the CRISPR/Cas9 system to generate leukemogenic chromosomal translocations in vivo. We generated two types of translocations in bone marrow cells with frequencies of 0.6% and 0.16%. Although the generation of *Tcf3-Hlf* was not sufficient to induce leukemia in this study the model can now be used to evaluate different cells of origin and to screen for required cooperative genetic events. Our approach can be employed to model any pair of genes as long as the orientation to the centromere does not result in dicentric chromosomes. Using the inbred Cas9 mouse strain will further simplify this approach. Besides, our vector design facilitates the combination of multiple sgRNAs to introduce additional oncogenic lesions (Supplementary Fig. 1B). Thus, our strategy provides an efficient way to generate precise translocations, enabling functional modeling in mice and constitutes a framework to study the role of cooperative events and the cells of origins in leukemogenesis.

## Supplementary Material

Supplemental Digital Content

## References

[1] Mertens F Johansson B Fioretos T The emerging complexity of gene fusions in cancer. *Nat Rev Cancer.* 2015;15:371–381. 25998716 10.1038/nrc3947

[2] Meyer C Burmeister T Groger D The MLL recombinome of acute leukemias in 2017. *Leukemia.* 2018;32:273–284. 28701730 10.1038/leu.2017.213 PMC5808070

[3] Jacoby E Chien CD Fry TJ Murine models of acute leukemia: important tools in current pediatric leukemia research. *Front Oncol.* 2014;4:95. 24847444 10.3389/fonc.2014.00095 PMC4019869

[4] Almosailleakh M Schwaller J Murine models of acute myeloid leukaemia. *Int J Mol Sci.* 2019;20:453. 10.3390/ijms20020453 PMC6358780 30669675

[5] Forster A Pannell R Drynan LF Engineering de novo reciprocal chromosomal translocations associated with Mll to replicate primary events of human cancer. *Cancer Cell.* 2003;3:449–458. 12781363 10.1016/s1535-6108(03)00106-5

[6] Brunet E Jasin M Induction of chromosomal translocations with CRISPR-Cas9 and other nucleases: understanding the repair mechanisms that give rise to translocations. *Adv Exp Med Biol.* 2018;1044:15–25. 29956288 10.1007/978-981-13-0593-1_2 PMC6333474

[7] Blasco RB Karaca E Ambrogio C Simple and rapid in vivo generation of chromosomal rearrangements using CRISPR/Cas9 technology. *Cell Rep.* 2014;9:1219–1227. 25456124 10.1016/j.celrep.2014.10.051

[8] Maddalo D Manchado E Concepcion CP In vivo engineering of oncogenic chromosomal rearrangements with the CRISPR/Cas9 system. *Nature.* 2014;516:423–427. 25337876 10.1038/nature13902 PMC4270925

[9] Oldrini B Curiel-Garcia A Marques C Somatic genome editing with the RCAS-TVA-CRISPR-Cas9 system for precision tumor modeling. *Nat Commun.* 2018;9:1466. 29654229 10.1038/s41467-018-03731-w PMC5899147

[10] Rajan SS Li L Kweh MF CRISPR genome editing of murine hematopoietic stem cells to create Npm1-Alk causes ALK(+) lymphoma after transplantation. *Blood Adv.* 2019;3:1788–1794. 31189527 10.1182/bloodadvances.2018025247 PMC6595257

[11] Reimer J Knoss S Labuhn M CRISPR-Cas9-induced t(11;19)/MLL-ENL translocations initiate leukemia in human hematopoietic progenitor cells in vivo. *Haematologica.* 2017;102:1558–1566. 28572162 10.3324/haematol.2017.164046 PMC5685230

[12] Andersson AK Ma J Wang J The landscape of somatic mutations in infant MLL-rearranged acute lymphoblastic leukemias. *Nat Genet.* 2015;47:330–337. 25730765 10.1038/ng.3230 PMC4553269

[13] Platt RJ Chen S Zhou Y CRISPR-Cas9 knockin mice for genome editing and cancer modeling. *Cell.* 2014;159:440–455. 25263330 10.1016/j.cell.2014.09.014 PMC4265475

[14] Zhu S Li W Liu J Genome-scale deletion screening of human long non-coding RNAs using a paired-guide RNA CRISPR-Cas9 library. *Nat Biotechnol.* 2016;34:1279–1286. 27798563 10.1038/nbt.3715 PMC5592164

[15] Huang Y Mouttet B Warnatz HJ The Leukemogenic TCF3-HLF complex rewires enhancers driving cellular identity and self-renewal conferring EP300 vulnerability. *Cancer Cell.* 2019;36: 630-644 e639. 10.1016/j.ccell.2019.10.004 31735627

[16] Fischer U Forster M Rinaldi A Genomics and drug profiling of fatal TCF3-HLF-positive acute lymphoblastic leukemia identifies recurrent mutation patterns and therapeutic options. *Nat Genet.* 2015;47:1020–1029. 26214592 10.1038/ng.3362 PMC4603357

[17] Nutt SL Heavey B Rolink AG Commitment to the B-lymphoid lineage depends on the transcription factor Pax5. *Nature.* 1999;401:556–562. 10524622 10.1038/44076

[18] Duque-Afonso J Smith KS Cleary ML Conditional expression of E2A-HLF induces B-Cell precursor death and myeloproliferative-like disease in knock-in mice. *PLoS One.* 2015;10:e0143216. 26588248 10.1371/journal.pone.0143216 PMC4654581

