# Supplementary material for: Rapid Generation of Leukemogenic Chromosomal Translocations in Vivo Using CRISPR/Cas9
Source: Hemasphere. 2020 Sep 30;4(5):e456. doi: 10.1097/HS9.0000000000000456 (PMC7544329; doi:10.1097/HS9.0000000000000456)
Supplement: Supplemental Digital Content [file hs9-4-e456-s001.docx]

**Supplemental Methods**

**Animals**

The *Cas9GFP* knockin mouse (Gt(ROSA)26Sor ^tm1.1(CAG-cas9*,-EGFP)Fezh^) was characterized previously.[^1^](#_ENREF_1) The Pax5 heterozygous (*Pax5^+/-^*) mouse was provided by Prof. Meinrad Busslinger (Research Institute of Molecular Pathology, Austria).[^2^](#_ENREF_2) The *Cas9GFP* and C57BL/6 mice (*Pax5^+/+^* or *Pax5^+/-^*) were crossbred to generate the *Cas9^+/-^ Pax5^+/+^* or *Cas9^+/-^ Pax5^+/-^* mice. Mice were housed and bred under pathogen-free conditions in our local animal facility (University of Zurich). All animal experiments were approved by the veterinary office of the Canton of Zurich, Switzerland.

**Vector construction**

An optimized lentiviral vector coding sgRNA and RFP657 (sg_shuttle_RFP657, Addgene 134968) was assembled with the U6-sgRNA cassette followed by EFS-RFP657. A special design of the restriction sites flanking the U6-sgRNA cassette facilitates the combination of multiple single sgRNA cassettes from each vector into one vector. Individual sgRNAs targeting translocation associated loci were designed using CRISPOR[^3^](#_ENREF_3) and cloned by annealing 2 DNA oligonucleotides and ligating into BsmBI-digested sg_shuttle_RFP657 vectors. After assessment of the editing efficiencies of each sgRNA, the 2 selected sgRNAs were then combined together into one vector (see details in Figure S1). All sgRNA sequences used in this study are listed below.

sg_Kmt2a (1): GTGTTAGCCATTAAACTTGG

sg_Kmt2a (2): ATCTAAGATTTAACAGGGCA

sg_Kmt2a (3): TCAGAAGTATAGCCTGGCTA

sg_Mllt1 (1): CCCAGCAGCAAAAAACGGAG

sg_Mllt1 (2): GCCAGACCAGCACTGATGAG

sg_Mllt1 (3): AGTGTTAAGACACTTGCAGG

sg_Tcf3: GAATGACATGTCAGGGTGGC

sg_Hlf: AAGCCTACATGTGTACTCAT

**Lentivirus production and transduction**

Lentivirus were produced by transfecting HEK293T cells with sgRNA vector (sg_shuttle_RFP657), psPAX2 (Addgene, 12260) and pVSV.G (Addgene, 8454) in a ratio of 5:3:2 using polyethylenimine (Polysciences, 24765-2). Virus was collected 30 hours after transfection. Cells were then exposed to crude virus for 24 hours followed by three times washing with phosphate-buffered saline (PBS).

**Assessment of the editing efficiency of sgRNA**

The editing efficiencies of each sgRNA were monitored in NIH 3T3 cells using qPCR-based assessment.[^4^](#_ENREF_4) Briefly, lentiviral vectors coding individual sgRNA (sg_shuttle_RFP657) were transduced into NIH 3T3 cells expressing Cas9. Four days after transduction, genomic DNA were harvested for qPCR detection using primers whose 3’end were overlapping with CRISPR targeting sites. The relative amplification efficiencies of targeting region primers to control region primers were calculated. The sequences of testing primers used in this study are listed below.

Kmt2a.sg1_F: GTGTAGGTGTCCCTATCTGGGAG

Kmt2a.sg1_R: TGTTCTTCCCAAGGACCTCCAAG

Kmt2a.sg2_F: GACGTGTGTTACACCATGCCC

Kmt2a.sg2_R: AGCAACACAGAGTTCAGGCAAC

Kmt2a.sg3_F: GGTGTGCACCACCCTAGCC

Kmt2a.sg3_R: CCACTGTGTTAACCCGATGCAG

Mllt1.sg1_F: CATGTAAAAAGGCTGGGGACATG

Mllt1.sg1_R: CCCCAGCAGCAAAAAACGGA

Mllt1.sg2_F: GAGCCAGACCAGCACTGATGA

Mllt1.sg2_R: CCAACTAGAATCCCTGCAGTGTG

Mllt1.sg3_F: GAGTACAGTGTTAAGACACTTGCAG

Mllt1.sg3_R: GAGCCTGAGACCTTGAAGAGC

**Genotyping**

Genomic DNA and RNA was extracted from transduced cells using a DNeasy Blood & Tissue kit (QIAGEN) and an RNeasy Mini kit (QIAGEN). cDNA was synthesized using a High-Capacity cDNA Reverse Transcription kit (Applied Biosystems). The sequences of primers for genotyping used in this study are listed below. The junctions of the translocation were verified by sequencing.

K.e10_F: GTGTGGGAGATGGGAGGCTTAG

K.e11_R: CCTGATGCTGTCTTCCACACAC

K.i10_F: GGATATTTGCCTATACCTATACCTAAAGAG

K.i10_R: CCACTGTGTTAACCCGATGCAG

M.e1_F: GCGCCAGTCATGGACAATCAG

M.e2_R: GCTTGGGTTTAGGGAAGCTGTC

M.i1_F: CAGTTGCAGCCCATCACTGAG

M.i1_R: GCACAAAGCCACTACACTCCAAG

T.e14_F: GAGCGCCCAGTGCCTTATC

T.e15_F: CTTGGACGAGGCCATCCATGTC

T.e16_R: CACTATAGGAGTCGGGAGGTCTCTG

H.e3_F: GCAATACACCGAGTCCCATTGAC

H.e4_R: CCTGGCCTCGTACTTGGCAAG

H.i3_F: GCATGGTGGTGCTAAGGAGTCAATG

H.i3_R: GCCATGGCTTTGGTCCTAGAG

**Bone marrow transduction and transplantation**

The *Cas9GFP ^+/-^* C57BL/6 haploid mice were generated by crossbred the *Cas9GFP* knockin mice (Gt(ROSA)26Sor ^tm1.1(CAG-cas9*,-EGFP)Fezh^)[^1^](#_ENREF_1) with C57BL/6 mice (wild type or *Pax5^+/-^*)[^2^](#_ENREF_2). Total bone marrow cells were isolated from femurs and tibias from 8- to 12- week-old *Cas9GFP^+/-^* C57BL/6 haploid mice. Bone marrow cells were cultured in StemSpan SFEM (StemCell Technologies) supplemented with 50 ng/ml murine Thpo (Peprotech), 50 ng/ml murine Scf (Peprotech) and 5 uM Cyclosporin A. Fresh prepared lentiviral supernatant was added immediately into cell cultures. Twenty-four hours after incubation with virus, cells were harvested, washed with PBS and intravenously injected into lethally irradiated (5 Gy x2, separated by 4 hours) 10- to 12- week-old C57BL/6 mice (10 million cells/mouse). An aliquot of transduced cells was kept to culture for additional days in StemSpan SFEM supplemented with 50 ng/ml murine Thpo and 50 ng/ml murine Scf for *in vitro* assessment.

Transplanted mice were strictly monitored twice per week according to a scoresheet that was approved by the authorities. Observational parameters include behavior, pain, fur appearance and body weight. Peripheral blood from mice was sampled every 2-4 weeks through tail vein bleeding started at 4 weeks after transplantation to monitor the engraftment of donor cells (GFP^+^) by flow cytometry. Mice were sacrificed when succumbed to moribund or presented body weight loss greater than 20%.

**Detection of translocation events by digital PCR**

The copy numbers of chromosomal translocations induced by paired sgRNAs in bone marrow cells were quantified using Droplet Digital PCR (ddPCR; Bio-Rad QX200) by Microsynth AG (Switzerland). Four days after transduction of sgRNA vectors, genomic DNA from *ex vivo* cultured bone marrow cells was extracted using a DNeasy Blood & Tissue kit (QIAGEN) and purified using an OneStep PCR Inhibitor Removal kit (Zymo Research, D6030). Triplicates of 20ul reactions contained 10ul 2×Supermix EvaGreen (Bio-Rad,1864034), 200nM of each primers and 50ng (control groups) or 75ng (sgRNA transduced groups) genomic DNA. For the detection of translocations, additional spike-in controls were set up as control gDNA spike-in with 48 aM synthetic artificial dsDNA templates of the translocations. The sequences of artificial templates of translocations are listed below. ddPCR program was carried out using following conditions: 5min at 95°C for the enzyme activation followed by 50 cycles of denaturation at 95°C for 30 sec and annealing/extension for 1 min at optimized temperature for each assay. Afterwards, signal was stabilized by applying 4°C for 5min and then 90°C for 5 min. Optimized annealing/extension temperatures (56 °C for the detection of *Kmt2a* and *Kmt2a-Mllt* and 64 °C for the detections of *Tcf3* and *Tcf3-Hlf*) were determined by pilot gradient PCR test. The results were analyzed with QuantaSoft™ Analysis Pro software.

Artificial templates of translocations:

*Kmt2a-Mllt1*

GGATATTTGCCTATACCTATACCTAAAGAGATATTTGAGAGAGGCATAAAAAATAAAATTTATTTTACTTCATGTATTGTTATACATATCTCAAAAGTAATCTTAGACAATAGTTTTTTTGAGACAGGAACTCACTTTGTAGACCAGGCTGGCCTCAGATTCACAGAGTGCCACCTGCCTCTCTCTACTGCTGGGATCAAAGGTGTGCACCACCCTAGGAGAGGCCCTTCGTCAATATGTACAGTGAAAAGTTTGGGGGGAGGATCTTTGTGCTTGCATACATGAATATAACATGTCCAGAGAAGGTTTTACACACTGCAGGGATTCTAGTTGGCTCCCTTTTCTGAGGTGGGCCCACCTTTGTGCTGGGAATCCCTTGGAGTGTAGTGGCTTTGTGC

*Tcf3-Hlf*

CTTGGACGAGGCCATCCATGTCCTGCGAAGCCACGCTGTTGGCACCGCTAGCGATCTCCATGGGCTTTTGCCTGGCCATGGCGCACTGACCACGAGCTTCACCGGCCCCATGTCACTGGGCGGGCGGCATGCCGGCCTGGTGAGTATCAGCTTAGGTCAGGGAATGGTGACATTTAGGGACTCCAGCCCATAGGATTAAAGTAAGTTAAAAAAGAAAAGTTCAACAAAGAAAGTGAAGAAAATTGGAATGGAATTTAGGGAGTGTGGTGAGCCTTTGGCATTCAGCTGAACTCTCCGCTCTCCTGTACTGGCACACAACTGCATTGGGCAAAGCCTGTTGCGACAGGTTGGTCCTTTCTCTAGGACCAAAGCCATGGC

**Flow cytometry**

To detect lineage markers, cells were stained with PE anti-mouse CD3 (BioLegend 100308), PE/Cy7 anti-mouse B220 (BioLegend, RA3-6B2), APC/Cy7 anti-mouse Gr-1 (BioLegend, RB6-8C5), APC anti-mouse CD11b (BioLegend, 101212), PE/Cy7 anti-mouse Sca-1 (BioLegend, 108114) and APC anti-mouse c-Kit (BioLegend, 105812).

**Fluorescence in situ hybridization (FISH)**

FISH was carried out on metaphase spreads prepared from spleen cells of the leukemia mice using whole chromosome paints (AMP09G and AMP17R from Cytocell) according to the manufacturer’s instruction.

**References**

1. Platt RJ, Chen S, Zhou Y, et al. CRISPR-Cas9 knockin mice for genome editing and cancer modeling. *Cell*. 2014;159(2):440-455.

2. Nutt SL, Heavey B, Rolink AG, Busslinger M. Commitment to the B-lymphoid lineage depends on the transcription factor Pax5. *Nature*. 1999;401(6753):556-562.

3. Haeussler M, Schonig K, Eckert H, et al. Evaluation of off-target and on-target scoring algorithms and integration into the guide RNA selection tool CRISPOR. *Genome Biol*. 2016;17(1):148.

4. Yu C, Zhang Y, Yao S, Wei Y. A PCR based protocol for detecting indel mutations induced by TALENs and CRISPR/Cas9 in zebrafish. *PLoS One*. 2014;9(6):e98282.

**Supplemental Figures**

**
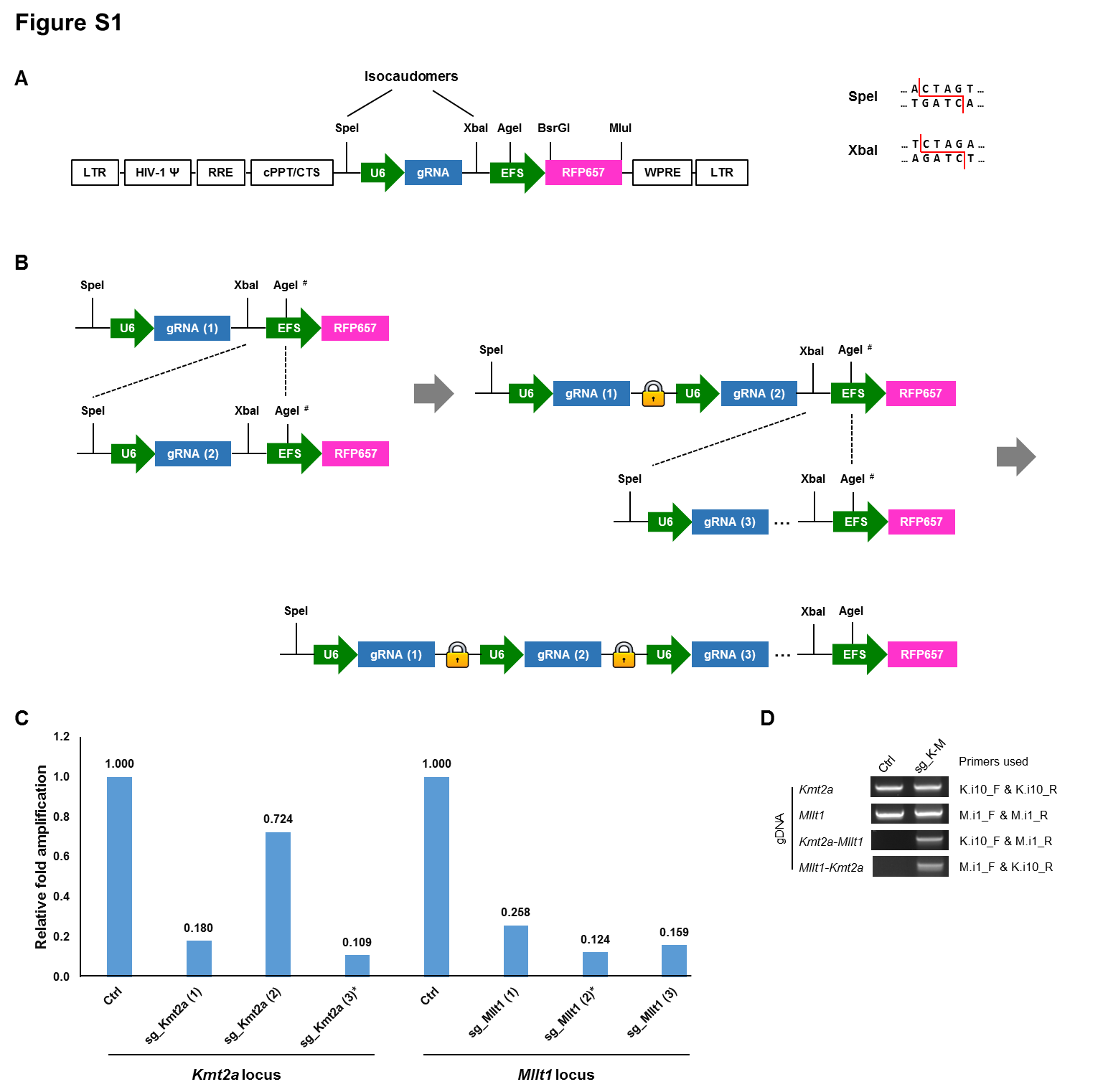
**

**Figure S1.** (A) Schematic of an optimized lentiviral vector coding sgRNA and RFP657 (sg_shuttle_RFP657) with a special design of the isocaudomers sites flanking the U6-sgRNA cassette. (B) Cloning strategy of combining multiple single U6-sgRNA cassettes from individual vectors into one vector using the isocaudomer sites. (C) Editing efficiencies of individual sgRNAs targeting *Kmt2a* and *Mllt1* loci in NIH 3T3 cells were monitored by qPCR as described previously[^4^](#_ENREF_4). Briefly, lentiviral vectors coding individual sgRNA were transduced into NIH 3T3 cells expressing Cas9. Four days after transduction, genomic DNA from sorted transduced cells (RFP657^+^) was harvested for qPCR detection using primers whose 3’end were overlapping with CRISPR targeting sites. The relative amplification efficiencies of targeting region primers to control region primers were presented. (D) PCR genotyping on genomic DNA of NIH 3T3 cells transduced with or without sgRNA vectors (sg_K-M) using primers indicated in Figure 1B.

^#^ Other unique restriction sites downstream of AgeI (eg. BsrGI or MluI) can also be used for cloning.

* The 2 selected sgRNAs that were combined into to one vector and used to generate *Kmt2a*-*Mllt1* translocation.

**
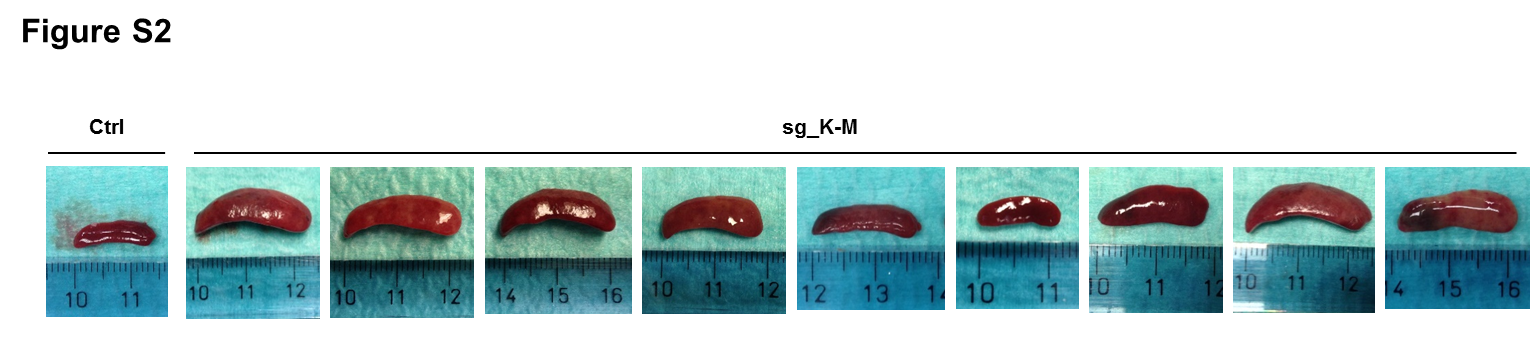
**

**Figure S2. Spleens from control and *Kmt2a-Mllt1* leukemic mice.**

**
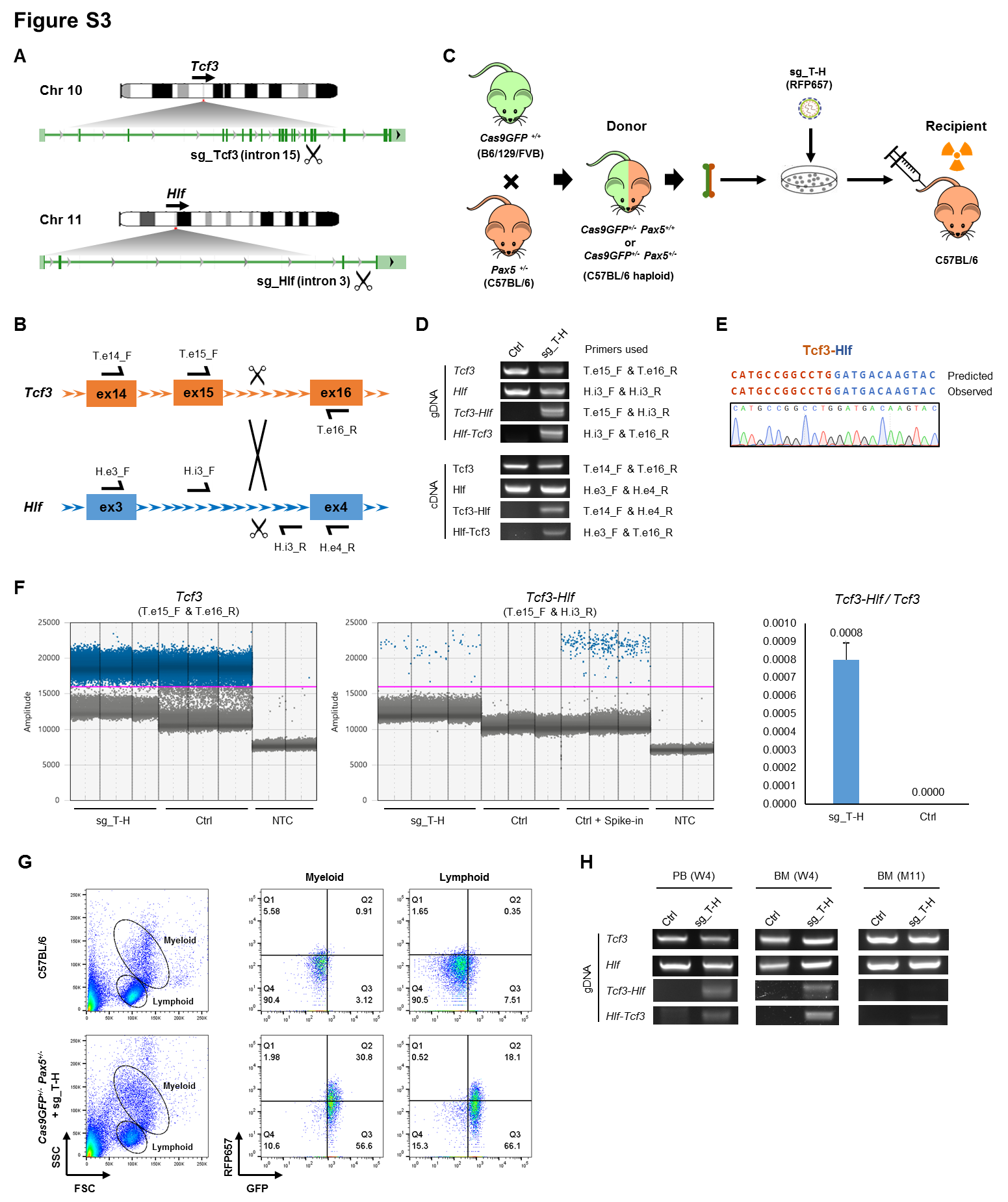
**

**Figure S3. Generation of *Tcf3-Hlf* rearrangement in murine hematopoietic cells using the CRISPR/Cas9 system.** (A) Schematic of the t(10;11) translocation involving the *Tcf3* and *Hlf* loci. Introns targeted by sgRNAs are indicated. (B) Schematic of the *Tcf3* and *Hlf* loci with the location of the primers used for genotyping. (C) Strategy of induction of *Tcf3-Hlf* rearrangement in murine bone marrow cells (*Pax5^+/+^* or *Pax5^+/-^*) and transplantation. (D) PCR genotyping of cultured bone marrow cells from a *Cas9GFP^+/-^ Pax5^+/-^* mice transduced with or without sgRNA vectors (sg_T-H). PCRs were performed on genomic DNA (gDNA) or cDNA from bone marrow cells *ex vivo* cultured for 4 days after transduction. (E) Sequence of the PCR product from cDNA showing the Tcf3-Hlf junction. (F) ddPCR quantification of *Tcf3* and *Tcf3-Hlf* frequencies in gDNA from bone marrow cells *ex vivo* cultured for 4 days after transduction of sgRNA vectors. Signal amplitudes of amplicon‐positive (blue) and ‐negative (gray) droplets for the detection of *Tcf3* and *Tcf3-Hlf* (left and middle) and the copy number ratio of *Tcf3-Hlf* with respect to *Tcf3* in Ctrl and sgRNA transduced groups (right, data are presented as mean with SD). Primer pairs for detections are indicated in the parenthesis; NTC, no template control; Ctrl+Spike-in, control gDNA spike-in with synthetic artificial DNA templates of *Tcf3-Hlf*. (G) Flow cytometry analysis of peripheral blood cells from a wild type C57BL/6 mouse and a mouse transplanted with bone marrow cells (*Cas9GFP^+/-^ Pax5^+/-^*) transduced with sg_T-H vectors at 4 weeks after transplantation. (H) PCR genotyping of engrafted cells from mice transplanted with bone marrow cells (*Cas9GFP^+/-^* *Pax5^+/-^*) transduced with or without sgRNA vectors (sg_T-H). PCRs were performed on gDNA from peripheral blood cells (PB) or bone marrow cells (BM) harvested at 4 weeks (W4) or 11 months (M11) after transplantation.


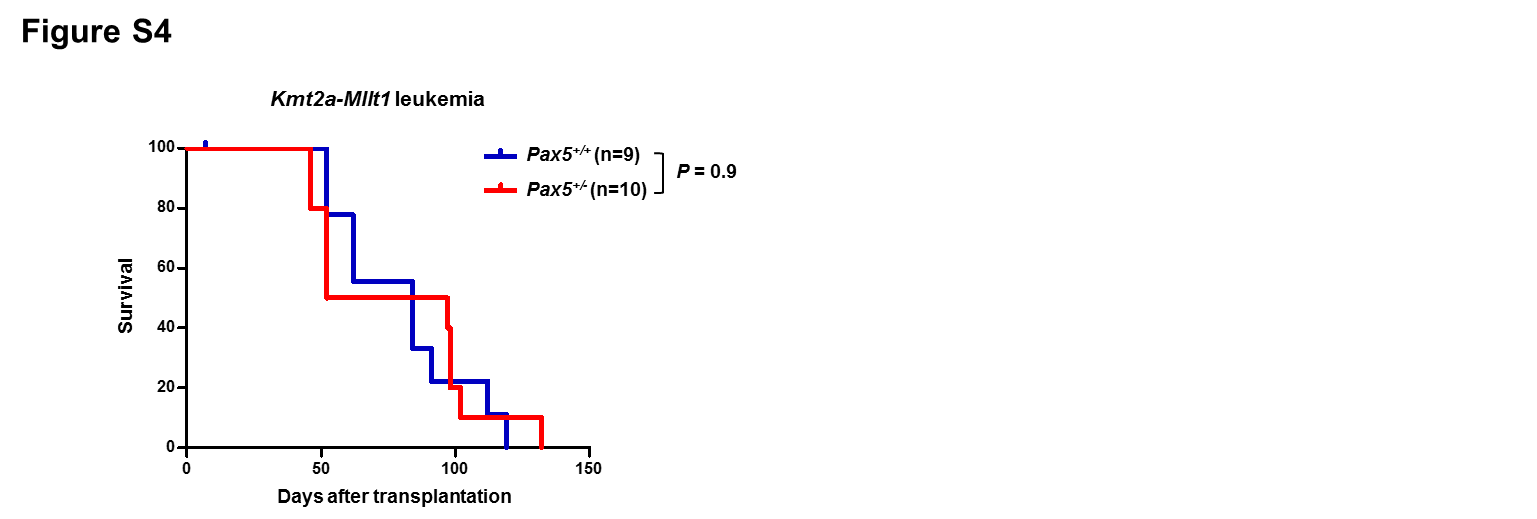


**Figure S4. The *Kmt2a-Mllt1* translocations induce leukemia with similar dynamics in the *Pax5^+/-^* background as in the *Pax5^+/+^* background**. Kaplan-Meier survival curves of mice transplanted with *Cas9GFP^+/-^* bone marrow cells (*Pax5^+/+^* or *Pax5^+/-^*) transduced with sg_K-M vectors developing *Kmt2a-Mllt1* leukemia. P value was determined by Mantel-Cox test.
